# A gene selection method for GeneChip array data with small sample sizes

**DOI:** 10.1186/1471-2164-12-S5-S7

**Published:** 2011-12-23

**Authors:** Zhongxue Chen, Qingzhong Liu, Monnie McGee, Megan Kong, Xudong Huang, Youping Deng, Richard H Scheuermann

**Affiliations:** 1 Biostatistics Epidemiology Research Design Core, Center for Clinical and Translational Sciences, The University of Texas Health Science Center at Houston, Houston, TX 77030, USA; 2 Department of Computer Science, Sam Houston State University, Huntsville, Texas 77341, USA; 3 Statistical Science Department, Southern Methodist University, Dallas, TX 75275, USA; 4 Department of Pathology, University of Texas Southwestern Medical Center, Dallas, TX 75390, USA; 5 Conjugate and Medicinal Chemistry Laboratory, Division of Nuclear Medicine and Molecular Imaging, Department of Radiology, Brigham and Women's Hospital and Harvard Medical School, Boston, MA 02115, USA; 6 Rush University Cancer Center, Rush University Medical Center, Chicago, IL 60612, USA

## Abstract

**Background:**

In microarray experiments with small sample sizes, it is a challenge to estimate p-values accurately and decide cutoff p-values for gene selection appropriately. Although permutation-based methods have proved to have greater sensitivity and specificity than the regular t-test, their p-values are highly discrete due to the limited number of permutations available in very small sample sizes. Furthermore, estimated permutation-based p-values for true nulls are highly correlated and not uniformly distributed between zero and one, making it difficult to use current false discovery rate (FDR)-controlling methods.

**Results:**

We propose a model-based information sharing method (MBIS) that, after an appropriate data transformation, utilizes information shared among genes. We use a normal distribution to model the mean differences of true nulls across two experimental conditions. The parameters of the model are then estimated using all data in hand. Based on this model, p-values, which are uniformly distributed from true nulls, are calculated. Then, since FDR-controlling methods are generally not well suited to microarray data with very small sample sizes, we select genes for a given cutoff p-value and then estimate the false discovery rate.

**Conclusion:**

Simulation studies and analysis using real microarray data show that the proposed method, MBIS, is more powerful and reliable than current methods. It has wide application to a variety of situations.

## Background

Microarray technology has been successfully used by biological and biomedical researchers to investigate gene expression profiles at the genome-wide level. Usually, the sample sizes are small compared to the number of genes to be investigated, making estimation of standard error for statistical tests very inaccurate. Furthermore, thousands of hypotheses (one corresponding to each gene or set of genes, in general) are tested at once, which greatly increases the probability of Type I error. This problem is also called the "multiple comparison problem" in hypothesis testing. A very small cutoff p-value is then needed to avoid picking a large number of false positives (FP); however, the price of that decision is failing to find many true positives whose p-values are larger than the cutoff value. When the sample sizes are extremely small, the problem worsens because as the sample size decreases so do the detection power and the ability to estimate p-values.

When the sample sizes are large enough, even if the data across two conditions are not normally distributed, we can still use a two-sample t-test to estimate the p-value for each gene. In practice, to avoid the normal distribution assumption, we may also choose non-parametric (rank-based) or permutation-based procedures. However, when sample sizes are very small, the t-test is not reliable due to the poor estimation for variances; many genes will have small p-values only because their estimated variances are too small. Furthermore, the t-test method treats each gene independently and does not utilize information shared among them. To borrow information from other genes, modified t-test methods have been proposed [1,2]. The modified t-test statistic is:

(1) $$T_{i}=\frac{d_{i}}{se_{i}+s_{0}}$$

where *d_i _*is the difference of means under two conditions for gene *i*; *se_i _*is the estimated standard error for *d_i _*and *s*_0 _is a constant, which is used to avoid too large absolute values of regular t-statistics due to very small estimated standard errors.

When we use test statistics in (1), we will lose the information about the distribution of true nulls since we do not know the distribution of (1). To overcome this problem, permutation-based procedures have been proposed [2]. One extensively used method in microarray data analysis is called SAM for "Significance Analysis of Microarray" [2]. SAM uses test statistics in (1) and then permutes sample labels to estimate the p-value for each gene.

The absolute values of statistics in (1) are usually smaller than that of regular t-statistics. When sample sizes are extremely small, the total number of distinguished permutations is limited and, therefore, permutation-based methods, such as SAM, will have larger p-values than those from regular t-test, especially for differentially expressed (DE) genes. For example, in experiments where there are only three replicates for two conditions (a typical scenario) there exist only ten different available permutations. The coarseness of the possible selections creates a problem for finding a reasonable cut-off p-value.

To select DE genes, we use a cutoff p-value and pick those genes whose p-values are smaller than the given cutoff value. Understood in this process and in any gene selection is the trade-off between false positives (type I error) and false negatives (type II error). If we want to control family-wise error rate (FWER), we need a very small cutoff p-value that will fail to find many true positives. Some researchers have proposed a strategy of, instead of controlling FWER, controlling false discovery rate (FDR) to allow some FPs in the set of selected genes, but to control the mean of the ratio of number of FPs to the number of total declared DE genes [3-5]. To control FDR, we need to estimate the number and the distribution of true nulls, which is quite difficult. Since it is difficult to separate non-DE genes from DE genes when doing permutations, the resulting estimated number and the distribution of the p-values for true nulls may not be accurate. Although several improvements for SAM have been proposed [6-8], Qiu et al showed that the permutation-based methods may have large variance and, therefore, are not reliable [9]. Yang and Churchill have noticed the problem of permutation-based methods when applied to small microarray experiments [8].

As part of SAM, Storey's FDR-controlling method has been proven to be more accurate than Benjamini and Hochberg's procedure and has been used extensively in microarray data analysis [4]. They defined a quantity called q-value. Similar to p-value, "a q-value threshold can be phrased in practical terms as the proportion of significant features that turn out to be false leads" [5]. Its R package, "qvalue," is publicly available [10]. "qvalue" first estimates the q-value for each p-value (gene) based on all p-values and then calculates the cutoff p-value for a given cutoff q-value. Although the authors claimed that "qvalue" usually conservatively controls the FDR in that its true false discovery rate is smaller than the given cutoff q-value [11], Jung and Jang have found that it could also be anti-conservative for small cutoff q-values [12]. In some cases, when the given cutoff q-values are small, "qvalue" may select very few or no DE genes.

In this paper, we show that when sample sizes are extremely small, the t-test has poor performance in terms of sensitivity and specificity and SAM (and "qvalue") may not be applicable due to the difficulty of controlling FDR for GeneChip array data. To circumvent those problems, we propose a new model-based method we call model-based information sharing method (MBIS). To evaluate the performance of our new method, we compare it with others by using both simulation data and real data.

## Method

### Fold change, equal variance, and data transformation

The ratio of the expression levels across two conditions is called fold change (FC); it has been used in the early comparative experiments [13,14]. This criterion is arguable since, depending on the decision-makers, choosing cutoff FC is arbitrary. Furthermore, the FC method does not take into account the variability with gene expression measurements, or, even worse, it assumes that the variability for all expression measurements is the same, which is likely to be false for most gene expression experiments. However, FC criteria have their own advantages. First, they are biologically meaningful and easily interpreted. Second, more importantly, many studies have shown that FC-based methods, if used appropriately, outperform other methods [15-19].

One way to obtain equal variance from gene to gene is to transform the data, usually with a logarithmic transformation. After this transformation, a FC (log scale) can be calculated from the difference of means across two conditions. However, different data sets may require different variance-stabilization transformations. Several variance-stabilization and normalization transformation methods, which try to transform expression values to be equal variance and normally distributed for each gene, have been proposed [19-23].

### Model-based information sharing (MBIS)

MBIS makes the assumption that an appropriate data transformation is available and has been applied to the raw gene expression data. This transformation has furthermore stabilized the variance. Therefore, the variance for each gene is a constant, denoted by *s*^2^, after transformation. If we can estimate *s*^2 ^from data, then we can calculate p-value easily for each gene.

### Estimation of *s*^2^

Suppose there are *n*_1 _and *n*_2 _replicates for condition one and two, respectively, and *G *genes to be tested. Under the assumptions of normality and equal variance, the estimated variance from each individual gene is an unbiased estimate for *s*^2 ^and has a Chi-square distribution with degrees of freedom *n*_1 _+ *n*_2 _- 2. Therefore the average of the estimated variances from all genes is also an unbiased estimate for *s*^2^:

(2) $${\bar{s}}^{2}=\frac{1}{G}\overset{G}{\underset{i=1}{\sum }}{\bar{s}}_{i}^{2}$$

where ${\bar{s}}_{i}^{2}$ is the estimated variance from individual gene *i *and *G *is the number of genes. Then we use the square root of ${\bar{s}}^{2}$, $\bar{s}$, as the estimated standard variance for each gene. From the equal variance assumption, we can use a normal distribution to approximate the mean difference of non-DE genes:

(3) $$d\sim N\left(0,s^{2}\left(\frac{1}{n_{1}}+\frac{1}{n_{2}}\right)\right)$$

Based on this normal distribution, we calculate the p-value for gene *i*:

(4) $$p_{i}=2*\left(1-\Phi \left(\frac{\left|d_{i}\right|}{\bar{s}\sqrt{\frac{1}{n_{1}}+\frac{1}{n_{2}}}}\right)\right)$$

where *d_i _*is the difference of the means for gene *i *across two conditions and Φ(.) is the cumulative distribution function (CDF) of the standard normal distribution.

### Estimation of total number of non-DE genes *G*_0_

For a given value *μ *(0 <*μ *< 1), we count the number (*N_u_*) of genes with p-values greater than or equal to *μ*. Then an estimate of *G*_0 _is *N_μ_*/(1-*μ*). To reduce the influence of DE genes since they have relatively small p-values, a relatively large *μ *is preferable. We can also use a vector of *μ*'s and calculate the corresponding estimated ${Ḡ}_{0}$'s and then take their (weighted) mean as the final estimate for *G*_0_.

### Gene selection and estimations for false positives and FDR

For a given cutoff p-value, *p*_0_, we pick those genes with p-values smaller than *p*_0 _as DE genes. Suppose *S *genes are selected. Then we can estimate the number of false positives, $\overset{\wedge }{\mathrm{FP}}=G_{0}\times p_{0}$, and the false discovery rate, $\overset{\wedge }{\mathrm{FDR}}=G_{0}\times p_{0}∕S$.

### SAM, t-test and q-value

For the SAM method, we use the R package, SAMr [10], and choose different values for s0.perc (percentile of estimated se's): -1 (t-test only, i.e. s0 = 0 in (1)), 20, 40, 60, 80 and 100. SAM will calculate p-values by permutation. For the t-test method, we calculate p-values from the regular t-test statistics (i.e. s0 = 0 in (1)) without permutation. We then use the calculated p-values for each method as the input for R package "qvalue" and then get the output of selected DE genes with different preset q-values.

### Simulation design

To restrict ourselves to small experiments, we assume the sample sizes for both conditions are 3, 5 and 8. We simulate 10,000 genes with normal distributions for two conditions. For non-DE genes, we assume they are normally distributed with a mean equal to 0; for DE genes, their absolute mean difference is uniformly distributed: with three ranges representing different degrees of differential expression: *U*(1,3), low, *U*(3,6), middle, and *U*(6,9), high. We assume the standard deviations are uniformly distributed as *U*(1,*b*), where *b *is greater than or equal to one. In the ideal situation, i.e. equal variance, *b *= 1. However, even after trying several variance-stabilization transformations, sometimes this assumption may be too strong for real data, and we therefore choose different *b*'s in our simulations: *b *= 1, 1.5 and 2. In other words, we simulate data with equal or near equal variance. The proportion of DE genes among all genes may also affect the gene selection results; we then choose three levels of proportions: 0.1, 0.3 and 0.5 (i.e. the numbers of DE genes are 1000, 3000 and 5000, respectively). The output of selected genes from "qvalue" for each method with different preset cutoff q-values: 0.05, 0.10, 0.15, 0.20 and 0.25, are compared.

### Real data set

We use Affymetrix GeneChip data sets selected from the GSE2350 series [24], downloaded from the NCBI GEO database [25] to compare our new method with others. We use the first three samples from both "control" (GSM44051, GSM44052 and GSM44053) and "CD40L treatment" (GSM44057, GSM44058 and GSM44059) groups. For the raw intensity data, we use the "rma" function in R package "affy" [10] to do background correction, normalization, and summarization [26]. Then we apply different methods to the summarized expression values (already on log base 2 scale) to estimate p-values that are the input for the "qvalue."

To see which method gives more biologically meaningful results, we use the web-based tool, CLASSIFI algorithm [27-29], that uses Gene Ontology (GO) [30] annotation to classify groups of genes defined by gene cluster analysis using the statistical analysis of GO annotation co-clustering. We compare the median p-values of "topfile" from the output of CLASSIFI. In general, the smaller the p-value is, the more reasonable the results in terms of GO classification [27].

## Results

### Simulation results

**Figure 1:**
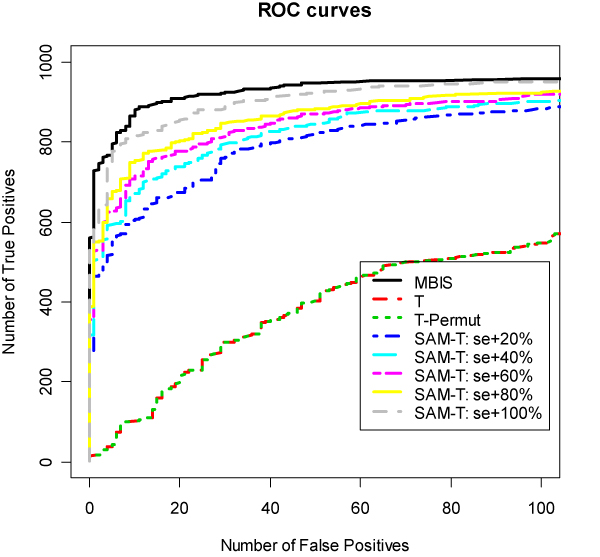
**ROC Curves**. ROC curves of MBIS, SAM with s0.perc = -1, 20, 40, 60, 80 and 100, and t-test from a simulated data set. There are three replicates for each condition. One thousand out of 10,000 genes are simulated differentially expressed with mean differences uniformly distributed between 3 and 6. The simulated variance for each gene is uniformly distributed between 1 and 1.5.

Figure 1 plots the Receiver Operating Characteristic (ROC) curves from different methods for our simulated data. The curves from regular t-test (without permutation) and SAM with s0 = 0 (T-Permut, i.e. t-test with permutation) are almost identical and perform worst in terms of sensitivity and specificity. Figure 1 clearly shows that information-sharing methods (SAM with s0>0 and MBIS) perform better. Our new method, MBIS, outperforms all SAM and t-test methods.

Table 1 gives the numbers of true positives (TP), false positives (FP), and the observed false discovery rates (Obs. FDR), FP/(FP+TP), obtained by "qvalue" with preset q-values: 0.05, 0.10, 0.15, 0.20 and 0.25, respectively, from a simulation. In this simulation, there are 1,000 DE genes out of 10,000 genes, three replicates for both conditions, *b *= 1.5, and the absolute mean differences for DE genes are uniformly distributed between three and six. For MBIS and t-test without permutation, we know the distribution of all nulls and, therefore, we can estimate the number of false positives (Est.FP) for a given cutoff p-value (calculated from given q-values by "qvalue"). As the ROC curves show, the regular t-test method performs more poorly than MBIS. For example, with preset q-value 0.05, the t-test method can only select 244 out of 1000 true positives at the price of 19 false positives. However, MBIS can obtain more than 95% true positives with only 94 false positives. Table 1 also shows that the numbers of estimated false positives from t-test and MBIS are very close to the true numbers of false positives, indicating that the estimated number and the distribution for true nulls are accurate for both the t-test and MBIS.

**Table 1:** Simulation results of numbers of TPs, and FPs from different methods (nde = 1000, rep = 3, b = 1.5, diff = c(3,6))

q-value		MBIS		SAM-T					
				
				S0 = 0	20	40	60	80	100
0.05	TP	957	244	0	0	0	0	0	0
	
	FP	94	19	0	0	0	0	0	0
							
	Est. FP	95	16						
	
	Obs. FDR	0.09	0.07	0	0	0	0	0	0

0.10	TP	976	669	0	0	0	0	0	0
	
	FP	203	99	0	0	0	0	0	0
							
	Est. FP	211	106						
	
	Obs. FDR	0.17	0.13	0	0	0	0	0	0

0.15	TP	983	821	0	771	835	821	877	891
	
	FP	324	228	0	16	26	16	27	26
							
	Est. FP	289	232						
	
	Obs. FDR	0.25	0.22	0	0	0.02	0.03	0.02	0.03

0.20	TP	992	896	474	893	910	909	917	932
	
	FP	488	379	44	80	92	81	85	75
							
	Est. FP	474	388						
	
	Obs. FDR	0.33	0.30	0.08	0.08	0.09	0.08	0.08	0.07

0.25	TP	994	924	704	916	926	929	935	949
	
	FP	632	529	116	145	142	134	141	129
							
	Est. FP	620	552						
	
	Obs. FDR	0.39	0.36	0.14	0.14	0.13	0.13	0.13	0.12

For the SAM methods with various s0.perc, when the preset q-value is small, we failed to get any true positives. For example, when given q-value 0.1, none of the SAM methods can get any true positives. Interestingly, when the given q-value is small, a regular t-test performs better than a t-test with a permutation in SAM; this implies permutation-based methods are not appropriate in this situation. Table 1 also indicates that SAM methods are usually conservative, as the authors of "qvalue" claimed [4]. However, it is not the case for MBIS and regular t-test. In general, the observed false discovery rates (Obs. FDR in Table 1) from MBIS and regular t-test methods are larger than the preset q-values, while SAM methods are usually too conservative and need large q-values to get a reasonable proportion of true positives. For different setups in our simulations, we obtained similar comparison results.

### Results from real data set

For the real data set, we use MBIS, regular t-test, and SAM to calculate the p-values for each gene and then use "qvalue" to select DE genes with cutoff q-values equal to 0.01, 0.025, 0.05, 0.075 and 0.1, respectively. By using "qvalue," we calculate the corresponding cutoff p-values from each cutoff q-value for these three methods. Since we know the distributions of nulls from MBIS and t-test (they have a uniform distribution for the p-values of nulls), and we can also estimate the number of true negatives for a given cutoff p-value, we can estimate the number of false positives and the false positive rates.

Table 2 summarizes the results. For a given cutoff q-value, the cutoff p-values calculated from "qvalue" for our new method and t-test are usually similar, but both are larger than that for SAM. Our new method usually selects more genes than the t-test does, which selects more genes than SAM does. In fact, for small cutoff q-values, for example, 0.01 and 0.025, SAM fails to select any genes due to the fact that the minimum of the estimated q-values from "qvalue" for SAM is 0.04, larger than 0.01 and 0.025. However, when the cutoff q-value increases to 0.05, the number of genes selected by SAM jumps to 3695. On the other hand, although the numbers of selected genes by our new method and the t-test increase as the cutoff q-values increase, as expected, the increments are more stable. All these observations are consistent with what we have observed in our simulations.

**Table 2:** Results from real data for given cutoff q-values

q-value		0.01	0.025	0.05	0.075	0.1
p- cutoff (from "qvalue")	MBIS	0.00685	0.0240	0.0617	0.108	0.162
	T	0.00144	0.0155	0.0613	0.123	0.192
	SAM	0	0	0.00741	0.0560	0.0969

# DE genes	MBIS	3075	4306	5550	6458	7276
	T	561	2402	4748	6345	7435
	SAM	0	0	**3695**	**4734**	**5335**

# common DE genes	MBIS, T	459	1954	3861	5261	6330
	MBIS, SAM	0	0	**3694**	**4734**	**5335**
	T, SAM	0	0	3327	4504	5228

Est. FDR	MBIS	0.0177	0.0443	0.0884	0.133	0.177
	T	0.0186	0.0468	0.0937	0.141	0.187

The selected gene sets from MBIS and the t-test are usually different. For example, when the cutoff q-value is equal to 0.05, MBIS and the t-test select 5550 and 4748 genes, respectively; the number of common genes by these two methods is 3694. In other words, about 1000 genes are selected by the t-test that are not in the list from the MBIS. However, SAM selected genes also usually selected by MBIS.

From the CLASSIFI output with cutoff q-value 0.05, the median p-values (-log10 scale) are 15.30, 7.05 and 6.01 for MBIS, SAM, and t-test, respectively, indicating that SAM performs better than the t-test but worse than MBIS in terms of co-clustering for genes with similar function according to GO.

Since the cutoff p-values from the same cutoff q-value are different for these three methods, we then use the same cutoff p-values for each method and compare their selected genes. Table 3 gives the comparisons with cutoff p-values equal to 0.05, 0.025, 0.01, 0.005, and 0.0025. The corresponding cutoff q-values obtained by "qvalue" are always larger for SAM than for t-test and MBIS. But the number of selected genes by SAM is much smaller than those by t-test, and MBIS for each given cutoff p-value. Again, for a given cutoff p-value, the gene sets selected by t-test and MBIS are different, while SAM still selects almost a subset of genes obtained by MBIS. The observed FDRs from the t-test and MBIS are always larger than those estimated from the "qvalue," a finding that is consistent with our observations in simulations. The median p-values (-log10 scale) from CLASSIFI are 16.32, 8.31, and 6.76 for MBIS, SAM, and t-test, respectively, when the cutoff p-value is 0.01, indicating that MBIS outperforms SAM that, in turn, performs better than the t-test.

**Table 3:** Results from real data for given cutoff p-values

p-value		0.05	0.025	0.01	0.005	0.0025
q-cutoff (from "qvalue")	MBIS	0.0422	0.0257	0.0132	0.00788	0.00468
	T	0.0446	0.0313	0.0210	0.0158	0.0122
	SAM	0.0738	0.0600	0.0556	0.0546	0.0544

# DE genes	MBIS	5290	4352	3383	2835	2383
	T	4355	3096	1849	1230	792
	SAM	**3613**	**2223**	**958**	**482**	**242**

# common DE genes	MBIS, T	3503	2411	1371	890	556
	MBIS, SAM	**3608**	**2223**	**958**	**482**	**242**
	T, SAM	3145	1870	767	396	202

Est. FDR	MBIS	0.0742	0.0451	0.0232	0.0138	0.00823
	T	0.0834	0.0586	0.0393	0.0295	0.0229

## Discussion

When sample sizes are small, information shared by genes is helpful and should be used. While t-test treats each gene independently, both SAM and MBIS, use information shared among genes. When the equal variance assumption in MBIS is met, the estimated variance for gene *i *in the t-test has a Chi-square distribution with degrees of freedom of *n*_1 _+ *n*_2 _- 2:

(5) $${\bar{s}}_{i}^{2}\sim s^{2}\chi^{2}\left(n_{1}+n_{2}-2\right)$$

The variance for ${\bar{s}}_{i}^{2}$ is:

(6) $$Var\left({\bar{s}}_{i}^{2}\right)=2s^{4}\left(n_{1}+n_{2}-2\right)$$

And the square of standard error estimated in t-test has variance:

(7) $$Var\left({\bar{se}}_{i}^{2}\right)=Var\left({\bar{s}}_{i}^{2}\left(\frac{1}{n_{1}}+\frac{1}{n_{2}}\right)\right)=2s^{4}\left(n_{1}+n_{2}-2\right)\frac{{\left(n_{1}+n_{2}\right)}^{2}}{n_{1}^{2}n_{2}^{2}}$$

However, (2) has a Chi-square distribution with degrees of freedom *G*(*n*_1 _+ *n*_2 _- 2), and its variance is:

(8) $$\text{var}\;\left({\bar{s}}^{2}\right)=\text{var}\;\left(\frac{1}{G}\overset{G}{\underset{i=1}{\sum }}{{\bar{s}}_{i}}^{2}\right)=\frac{1}{G^{2}}\overset{G}{\underset{i=1}{\sum }}\text{var}\;\left({{\bar{s}}_{i}}^{2}\right)=\frac{2}{G}s^{4}\left(n_{1}+n_{2}-2\right)$$

The square of standard error estimated for our new method is:

(9) $$Var\left({\bar{se}}^{2}\right)=Var\left({\bar{s}}^{2}\left(\frac{1}{n_{1}}+\frac{1}{n_{2}}\right)\right)=\frac{2}{G}s^{4}\left(n_{1}+n_{2}-2\right)\frac{{\left(n_{1}+n_{2}\right)}^{2}}{n_{1}^{2}n_{2}^{2}}$$

In a typical microarray experiment, the number of genes, *G*, is usually between 10K and 50K, indicating that the variance in (9) is very close to 0 and the estimated value in (2) is close to the true value; therefore a normal distribution is appropriate to approximate the mean differences of the true nulls.

In comparing (7) with (9), we can see that, while the regular t-test method gives a much larger variance for each estimated variance (each individual t-test will lose two degrees of freedom due to variance estimation), MBIS, a method that utilizes information among genes, has a more precise estimate for the common variance. Therefore, MBIS always outperforms the t-test.

On the other hand, the Chi-square distribution is right skewed, implying that its mean is larger than its median. If ${\bar{s}}_{i}^{2}$'s have a Chi-square distribution, they are more likely to have estimated values less than the mean (true value) than estimated values greater than the mean. In other words, ${\bar{s}}_{i}^{2}$ are more probable to underestimate than overestimate the constant variance. Therefore many true nulls may have very small p-values from a t-test only because they have small estimated standard errors. This explains why there are so many FPs from t-test in our simulations; and consequently t-test selects so many different DE genes than SAM and MBIS do in real data. Because of the same reason, adding a common number to each individual *se_i _*in (1) will potentially decrease the bias (for small s0.perc in SAM) and/or decrease the relative difference of estimated variances for most genes; therefore SAM usually improves the test statistics, although still not as favorably as MBIS. This explains why SAM performs better than t-test but worse than MBIS in terms of sensitivity and specificity.

When sample sizes are extremely small, as we mentioned before, SAM will have relatively larger p-values due to a limited number of permutations available, affecting the estimation of q-values by "qvalue". "qvalue" does not perform very well in this situation. For a given cutoff q-value, the corresponding cutoff p-value calculated by "qvalue" could be too large (as seen in the results from t-test and MBIS in simulation and real data) or too conservative (as in the results from SAM), a finding consistent with those from Jung and Jang [12].

Another difficulty for "qvalue" is that the number of selected genes can be very sensitive to the cutoff q-value, especially the very small preset q-value (see Table 2), that is desirable in practice; in this situation, SAM even performs worse than the regular t-test in terms of proportion of the DE genes selected. This raises the question of how to choose an appropriate q-value in practice to which there is no absolute answer. Sometimes, even for large q-values (as seen in the results from SAM in Table 1), the "qvalue" gives us a small proportion of true positives; on the other hand, we could select a large number of genes with a small q-value (as seen in the results from MBIS and t-test for real data in Table 2). We recommend that in this situation (small sample sizes), instead of using q-value only, one should choose a cutoff p-value to select DE genes first and then estimate FDR if desired.

Although we assume equal variance in the MBIS, we also evaluate this new method under situations when this assumption is violated. By simulation, we have shown that, when the variances of gene expressions are near constant, MBIS still outperforms both the t-test and SAM, making our method applicable in various situations.

From our experience, variances estimated from raw expression data are highly variable. We should transform data before applying MBIS. Several variance-stabilization and normalization transformation procedures, such as logarithm, Box-Cox transformation, generalized logarithm [19], variance stabilization [21] and data-driven Haar-Fisz transformation for microarrays (DDHFm) [22], are already available. In addition, choosing appropriate preprocessing procedures (background correction, normalization and summarization) is also very important for downstream analyses, including gene selection [16,26,31-34].

## Conclusions

For microarray data with extremely small sample sizes, a modified t-test like SAM performs better than a regular t-test in terms of sensitivity and specificity. However, to control FDR, for small preset q-values, SAM fails to select enough true positives and performs worse than the t-test. To circumvent this problem, we propose a model-based information sharing method (MBIS) that uses information shared by genes. We show, using both simulation and real microarray data, that this new method outperforms the t-test and SAM.

## Competing interests

The authors declare that they have no competing interests.

## Authors' contributions

ZC devised the basic idea of the new method and drafted the manuscript; QL participated in study design and manuscript preparation; MK participated in the analyses based on CALSSIFI; RHS participated in developing this new algorithm; MM, XH and YD assisted the study and co-wrote the manuscript. All authors read and approve the final manuscript.
